# Epigenetic Alterations in Cryopreserved Human Spermatozoa: Suspected Potential Functional Defects

**DOI:** 10.3390/cells11132110

**Published:** 2022-07-04

**Authors:** Wanxue Wang, Plamen Todorov, Cheng Pei, Mengying Wang, Evgenia Isachenko, Gohar Rahimi, Peter Mallmann, Vladimir Isachenko

**Affiliations:** 1 Department of Obstetrics and Gynaecology, Medical Faculty, Cologne University, 50931 Cologne, Germany; wangwanxuewwx@gmail.com (W.W.); chengpei314@gmail.com (C.P.); wmymed@yeah.net (M.W.); evgenia.isachenko@uk-koeln.de (E.I.); gohar.rahimi@uk-koeln.de (G.R.); perter.mallmann@uk-koeln.de (P.M.); 2 Institute of Biology and Immunology of Reproduction of Bulgarian Academy of Sciences, Tsarigradsko highway 73A, 1113 Sofia, Bulgaria; plamen.ivf@gmail.com

**Keywords:** assisted reproductive technology, cryopreservation, spermatozoa, alternative splicing, exon skipping

## Abstract

Background: Gene set enrichment analysis (GSEA) was conducted on raw data, and alternative splicing (AS) events were found after mRNA sequencing of human spermatozoa. In this study, we aimed to compare unknown micro-epigenetics alternations in fresh and cryopreserved spermatozoa to evaluate the effectivity of cryopreservation protocols. Methods: Spermatozoa were divided into three groups: fresh spermatozoa (group 1), cryoprotectant-free vitrified spermatozoa (group 2), and conventionally frozen spermatozoa (group 3). Nine RNA samples (three replicates in each group) were detected and were used for library preparation with an Illumina compatible kit and sequencing by the Illumina platform. Results: Three Gene Ontology (GO) terms were found to be enriched in vitrified spermatozoa compared with fresh spermatozoa: mitochondrial tRNA aminoacylation, ATP-dependent microtubule motor activity, and male meiotic nuclear division. In alternative splicing analysis, a number of unknown AS events were found, including functional gene exon skipping (SE), alternative 5′ splice sites (A5SS), alternative 3′ splice sites (A3SS), mutually exclusive exon (MXE), and retained intron (RI). Conclusions: Cryopreservation of spermatozoa from some patients can agitate epigenetic instability, including increased alternative splicing events and changes in crucial mitochondrial functional activities. For fertilization of oocytes, for such patients, it is recommended to use fresh spermatozoa whenever possible; cryopreservation of sperm is recommended to be used only in uncontested situations.

## 1. Introduction

Assisted reproductive technology (ART) has helped millions of couples to have babies and helps to protect the fertility of cancer patients [1].

Spermatozoa cryopreservation technology is the most popular preservation protocol for male fertility [2]. Although there have been great improvements in cryopreservation procedures and their intrinsic value has grown, frozen semen has not been routinely used for human reproduction [3]. The main disadvantage is the lower fertility rate in ART cycles when using frozen–thawed spermatozoa compared to ART cycles using fresh unfrozen semen [4,5]. Therefore, improving the survival of sperm under cryopreservation and the safety of frozen semen remain major challenges [2,6]. Regarding the question of whether cryopreservation of human spermatozoa is a technology that is safe enough, there is no positive answer.

Cryopreservation involves sudden osmotic and temperature changes that may lead to molecular disturbances in the sperm transcriptome and proteomics [7,8,9]. A thorough understanding of these molecular disorders during cryopreservation is essential in order to optimize current cryopreservation protocols [2]. Concerning epigenetics, earlier studies showed that cryopreservation alters the profiles of specific genes involved in key sperm functions, such as motility, capacitation, oocyte binding ability, and the acrosome reaction [10]. Although it has been considered that human spermatozoa are transcriptionally silent, findings in other species suggest that transcriptional activity is possible in different contexts [11]. These findings encourage further research focused on uncovering the underlying epigenetic instability of sperm affected by cryopreservation [12]. Transcriptomics has now allowed the identification of sperm transcriptomes that are quantitatively affected by cryopreservation in humans [3,8]. With the development of high-throughput sequencing technology, the bioinformatics changes of gametes and embryos after cryopreservation have attracted a great deal of research attention [3]. Potential biomarkers affecting spermatozoa motility, quantity, ultrastructure, and fertility after cryopreservation can be understood by proteomics sequencing [13,14]. It is also possible to study biological changes in the genome and transcriptome of spermatozoa by using different concentrations of cryoprotectant [15]. With the development of cryopreservation techniques, there was a recent sequencing study of the transcriptomic changes in spermatozoa using different cryopreservation methods (slow freezing and vitrification) [8]. The scientific development of cryopreservation also offers hope for the establishment of living biobanks, which could aid in disease modeling, organoid culture, and precision medicine for cancer treatment [16].

The epigenetic alterations of our study are highly concentrated on ATP-dependent microtubule motor activity and mitochondrial tRNA aminoacylation. Alterative splicing also became part of this study later as a result.

The toxicity of cryoprotectants can also be a risk factor for conventional cryopreservation and vitrification of spermatozoa. Conventional freezing is a method that uses slow freezing with cryoprotective agents (CPAs) [17,18]. Vitrification of spermatozoa is a technology that involves no ice formation in the cooling down and warming process, with less injury to the samples [19,20,21]. CPAs used for ART in most labs are permeable, including dimethyl sulfoxide (DMSO), ethylene glycol (EG), and glycerol [22,23]. In recent years, non-permeable cryoprotectants such as sucrose or other agents have played a role in osmotic balance [24,25].

Since spermatozoa are stored in liquid nitrogen during cryopreservation, and viruses can remain pathogenic for a long time in liquid nitrogen, the possibility of contamination needs to be considered, which in turn would affect the safety of gametes [26,27]. However, studies have shown that viral particles can be removed during semen washing [28]. It has also been reported that it is possible for viruses to survive in liquid nitrogen [6,29]. In fact, viruses, especially enveloped ones, present big problems in cryopreservation. Notably, two of the most common viruses, HIV and hepatitis, are enveloped viruses. For non-enveloped viruses, the question of cryopreservation a priori does not arise because they have lipid membranes, and thus are water free. The stability of these viruses is well known [30]. For these viruses, cryopreservation means only cooling (for example, by plunging them directly into liquid nitrogen) and nothing more. For cancer patients with cryopreserved testicular cells and tissues, the risk of reintroducing cancer cells after rewarming needs to be considered [31]. Hematological malignancies, in particular, are associated with a significantly increased risk of re-morbidity following cryopreservation and autologous transplantation [32,33].

There are multiple security issues in cryopreservation (toxicity, epigenetics stability, microbial contamination) (Supplementary Figure S1). Epigenetic alterations after cryopreservation of cells are an issue that is seldom discussed [1,9].

Gene set enrichment analysis (GSEA) was conducted on raw data, and alternative splicing (AS) events were found after mRNA sequencing of human spermatozoa. In this study, we aimed to compare unknown micro-epigenetics alternations in fresh and cryopreserved spermatozoa to evaluate the effectivity of cryopreservation protocols.

## 2. Materials and Methods

### 2.1. Sample Collection

In Supplementary Figure S2, there is a flowchart of the methodology of the whole study.

Except where otherwise stated, all chemicals were obtained from Sigma (Sigma Chemical Co., St. Louis, MO, USA). The study was conducted in accordance with the Declaration of Helsinki and approved by the Ethics Committee of the University Hospital of Cologne (application 01-106).

After informed consent, the ejaculates samples were obtained from 9 patients by masturbation after 2–7 days of sexual abstinence. After liquefaction, the semen analysis was performed to evaluate the spermatozoa parameters, according to the latest published guidelines of the World Health Organization guidelines in the sixth edition [34]. Only normozoospermic samples were collected in this study. All specimens had a minimum of 20 million spermatozoa/mL, 60% progressive motility, and 4% morphologically normal spermatozoa. All the samples were normal sperm and the patients’ parameters were not statistically different. These parameters are very comparable since the same three persons were used in three groups. Fresh, conventional, and vitrified, these three groups using the samples obtained from the same ejaculate of the same patient. And the same ejaculate sample in every group were then divided into three equal parts (in every group, n = 3).

Routine protocols were used [23,35,36]. The swim-up method was performed to collect spermatozoa. Swim-up was carried out using a culture medium (HTF; Irvine Scientific, Santa Ana, CA, USA) supplemented with 10 mg/mL of HSA (Irvine Scientific, Santa Ana, CA, USA). After liquefaction, 1 mL of semen sample was put into a sterile 15 mL conical centrifuge tube, and then 1.2 mL of culture medium was gently layered over it. The tube was inclined to an angle of about 45° to increase the surface area of the semen-medium interface and incubated at 37 °C for 1 h. Then, the tube was gently returned to the upright position and the uppermost 1 mL of medium was removed. This contained highly motile sperm cells. Next, the uppermost medium was resuspended into a 2 mL culture medium and centrifuged at 400x *g* for 5 min, and the supernatant was discarded. The collected sperm pellets were resuspended in the basic culture medium to obtain a concentration of 50 × 10^6^ spermatozoa/mL, and finally aliquoted into equal subsamples for the different experimental groups.

### 2.2. Experimental Design and Cryopreservation

The 9 samples were equally divided into three groups: fresh spermatozoa (group 1), cryoprotectant-free vitrified spermatozoa (group 2), and conventionally frozen spermatozoa (group 3).

The detailed protocol of cryopreservation is published [9,23,35,36]. The package for cryoprotectant-free vitrified spermatozoa (group 2) was used in form of 600 µm capillaries from hydrophobic material (Vitromed GmbH, Jena, Germany). Then, a micropipettor (Eppendorf, Hamburg, Germany) was used to aspirate 10 µL of spermatozoa suspension and dropped into a 35×10 mm Falcon Petri dish (Life Sciences, Durham, NC, USA). The capillaries one by one were filled with 10 µL of spermatozoa suspension by aspiration using an IVF pipette controller (Medical Technology GmbH, Bruckberg, Germany) [23]. Next, the capillary was put into a one-end-sealed straw (Medical Technology GmbH, Bruckberg, Germany) prepared by a heat-sealer. It was directly plunged into liquid nitrogen after sealing another end of the straw.

The conventional frozen spermatozoa (group 3) were manipulated by the same 0.25 mL straw as samples in group 2. It was filled with 10 µL of spermatozoa suspension in the capillary in each straw. Then, the straws with capillaries were placed horizontally in liquid nitrogen vapor (−160 °C, 10 cm over the liquid nitrogen surface), kept for 30 min, and finally put into the liquid nitrogen for 10~20 days.

### 2.3. Transmission Electron Microscopy

The fresh sperm and cryopreserved spermatozoa of each treatment group were fixed for 48 h and manipulated as in our previous study [9]. Ultrathin sections of 70 nm were cut using an ultramicrotome (Leica Microsystems, UC6, Jena, Germany) and a diamond knife (Diatome, Biel, Switzerland) and stained with 1.5% uranyl acetate for 15 min at 37 °C and Reynolds lead citrate solution for 4 min. Images were acquired using a JEM-2100 Plus Transmission Electron Microscope (JEOL) operating at 80 kV equipped with a OneView 4 K camera (Gatan). The number of spermatozoa in each group was determined in a sample of 100 spermatozoa from at least two sets of sections.

### 2.4. Sequencing, Data Extraction and Gene Set Enrichment Analysis of mRNA Expression

As described in our previous publication, we detected 9 RNA samples, which were used for library preparation with an Illumina compatible kit and sequencing by the Illumina platform [19]. The raw data of this project (in fq.gz format) can be found in the Sequence Read Archive of the National Center for Biotechnology Information (see Data Availability).

In order to avoid low-quality reads and reads with adapters from raw data, we filtered the raw reads and obtained clean reads. The filtering procedure was as follows: (1) remove reads containing adapters; (2) remove reads containing N > 10% (N represents bases that could not be determined); (3) remove low-quality reads. The Q score (quality value) of >50% of the bases of the read is ≤5.

Gene expression was investigated by the abundance of transcripts (sequencing counts) that mapped to genomes or exons. Read matter is proportional to genetic expression level, genetic size, and sequencing depth. Anticipating the variety of pieces per kilobase of transcript series per million base pairs sequenced (FPKM) is the most typical method of estimating gene expression levels, which takes into consideration the impact of both sequencing depth and gene size on fragment counts. Raw data (raw reads) in FASTQ format were first run via internal Perl scripts. Overall, the downstream evaluations were based on clean top-quality data. The GSEA tool (http://www.broadinstitute.org/gsea/index.jsp, accessed on 30 June 2022) was used, and Gene Ontology (GO), Kyoto Encyclopedia of Genes and Genomes (KEGG), Reactome, Disease Ontology (DO), and DisGeNET [37] information sets were used for GSEA independently.

### 2.5. Alternative Splicing Analysis and Exon Skipping Events Annotation in KEGG Pathway

Alternative splicing (AS) analysis was performed by rMATS software, which provides a statistical method of robust and flexible detection of differential AS from replicate RNA-seq data [38,39]. It identifies AS events corresponding to all major types of AS patterns and calculates the *p*-value and FDR. These types include exon skipping (SE), alternative 5′ splice sites (A5SS), alternative 3′ splice sites (A3SS), mutually exclusive exons (MXE), and retained introns (RI). Graphs of alternative splicing sites were created by rMATS and python with the rmats2sashimiplot package [40].

Enrichment analysis of KEGG pathways for exon skipping events was manipulated by KOBAS-i intelligence tools and the clusterProfiler package in R [41,42]. We used *p*-value and the inclusive level difference value (InclevelDifference in metadata Excel files) to quantify the fold changes of exon skipping events, and all significant events can be detected by running our raw data.

## 3. Results

The GSEA and AS metadata materials can be found in figshare [43].

### 3.1. Gene Set Enrichment Analysis in Human Spermatozoa Sequencing

For Gene Ontology enrichment by GSEA, conventional frozen spermatozoa (group 1) and fresh spermatozoa (group 2) were compared. There were 42/309 gene sets upregulated in group 1 and 1221/7257 gene sets upregulated in group 2, and 6036/7257 gene sets are upregulated in group 1. Gene set size filters (min = 15, max = 5000) resulted in filtering out 15,474/22,731 gene sets. The remaining 7257 gene sets were used in the analysis. A total of 1154 gene sets were enriched at a nominal *p*-value < 0.01 (see detailed results in the figshare metadata file SfvsFresh.Gsea.1631762876624.rpt).

In the comparison of vitrified spermatozoa (group 3) and fresh spermatozoa (group 1), 1371/7257 gene sets were significant. A total of 81 gene sets were significantly enriched at nominal *p*-value < 0.01 (see detailed results in the figshare metadata file VivsFresh.Gsea.1631762935542.rpt).

The key result of gene set enrichment evaluation is the enrichment score (ES), which shows the level to which a gene set is overrepresented at the top or bottom of a listing of genes. GSEA computes the ES by walking down the rated checklist of genes, increasing the running sum when a gene is in the gene set and decreasing it when it is not. The magnitude of the increment depends on the relevance of the gene to the specific phenotype. Thus, the ES is the maximum value from zero encountered when walking the list. A significant ES shows gene set enrichment at the top of the ranked list; a non-significant ES shows gene set enrichment at the bottom of the list.

**Figure 1:**
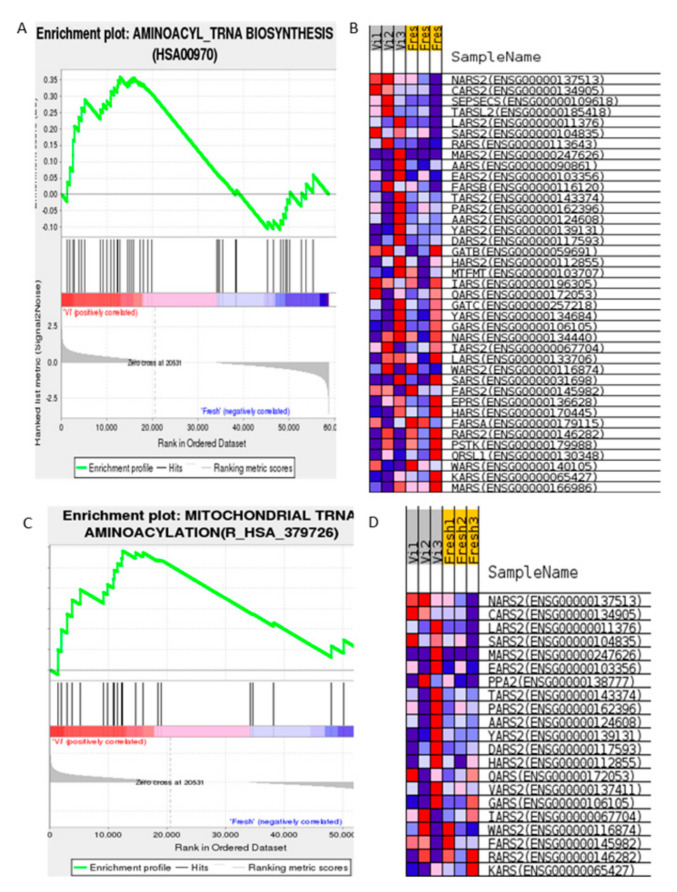
GSEA result for vitrified human spermatozoa: Aminoacylation process of tRNA in mitochondria. (**A**) Enrichment plot of aminoacylation term, GO ID: HSA00970. (**B**) Visualization of enriched genes in tRNA aminoacylation process in cryoprotectant-free vitrified spermatozoa and fresh spermatozoa groups. (**C**) Enrichment plot of mitochondrial tRNA aminoacylation term, Reactome ID: R_HSA379726. (**D**) Visualization of enriched genes in Reactome in cryoprotectant-free vitrified spermatozoa and fresh spermatozoa groups. Running enrichment score for gene set as analysis walks down scored list shown in (**A**). Enrichment score for gene set is value at top of graph (furthest from 0.0). Gene sets with a distinctive peak at the start. Center portion of plot reveals how members of gene set are distributed in scored list. Leading edge part of gene set indicates participants that contribute most to ES.

**Figure 2:**
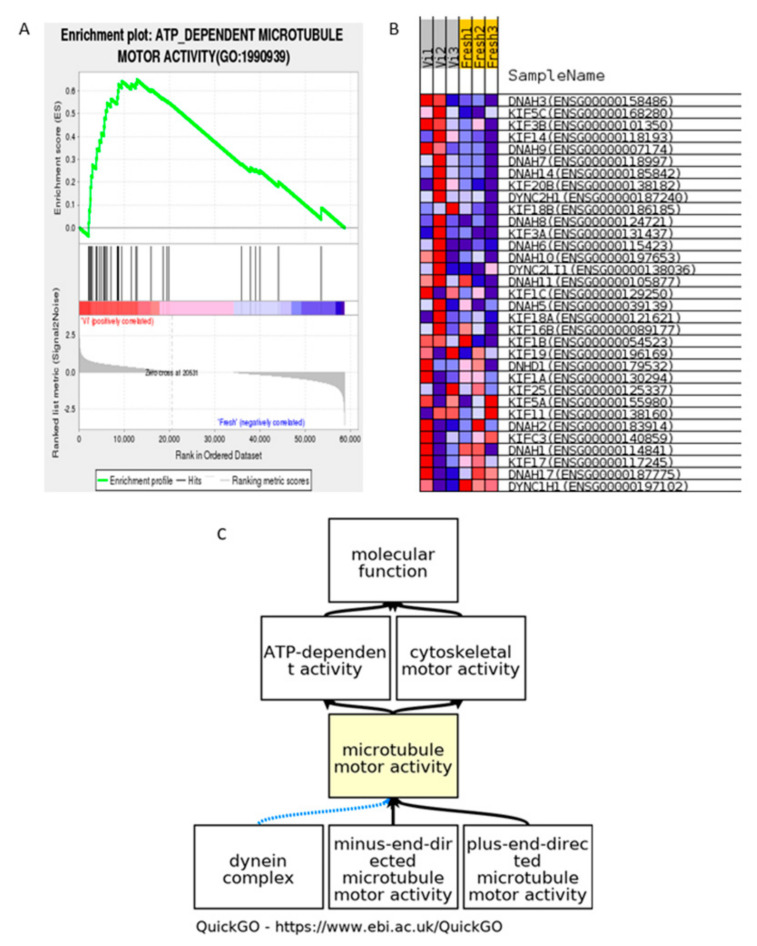
GSEA result of vitrified human spermatozoa: ATP-dependent microtubule motor activity (GO ID: HSA1990939). (**A**) Enrichment plot of microtubule motor activity. (**B**) Visualization of enriched genes in microtubule motor activity process in cryoprotectant-free vitrified spermatozoa and fresh spermatozoa groups. (**C**) Visualization of microtubule motor activity term in GO database. Valuable ES (Figure 2A): above edge part is set of members that show in rated list prior to peak rating. Non-valuable ES: set of members that show after peak score. Lower portion of graph shows condition of ranking statistics moving down list of rated genes. Ranking statistics indicate correlation between gene and an experimental group. Status of ranking metric changes from positive to non-significant moving down the list.

**Figure 3:**
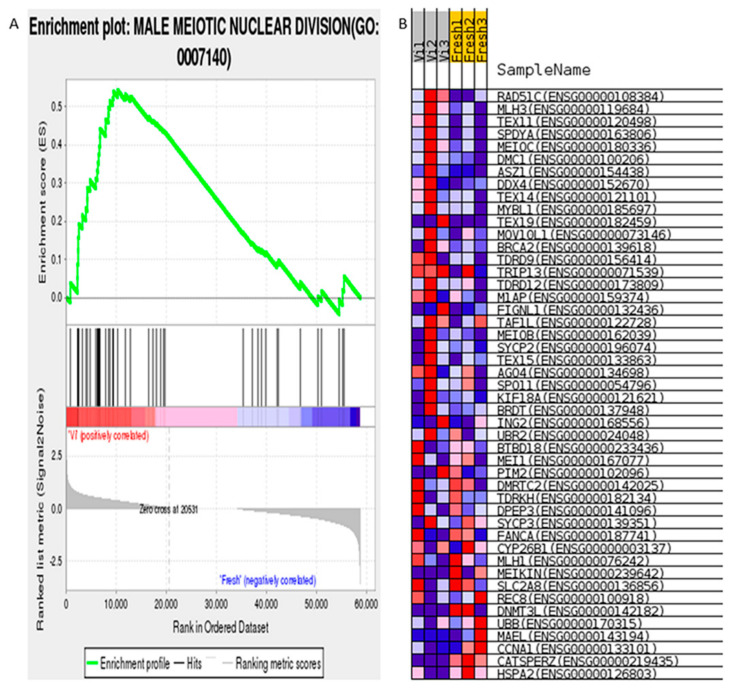
GSEA result of vitrified human spermatozoa: male meiotic nuclear division (GO ID:0007140). (**A**) Enrichment plot of male meiotic nuclear division. (**B**) Visualization of enriched genes in male meiotic nuclear division process in cryoprotectant free vitrified spermatozoa and fresh spermatozoa groups. Positive result indicates correlation with control group phenotype, non-significant value indicates relationship with experimental group ((**A**) and Figure 1A,C and Figure 2A; more examples can be seen in Figshare in file GSEA/1.GO/SfvsFresh/Sf/pos_snapshot.html) [43].

In the evaluation results, the enrichment graph gives a visual view of the gene set enrichment scores (Figure 1, Figure 2 and Figure 3).

#### 3.1.1. Gene Expression Set Analysis Shows Cryopreservation Process Is Significantly Relevant to Mitochondrial tRNA Aminoacylation in Human Spermatozoa

The gene set is enriched into two highly relevant terms: GO ID: HSA00970 and Reactome ID: R_HSA379726 (Figure 1). Significant impact genes are shown in Supplementary Figure S2 with ranking differences in signaling pathways indicated in red on the graph.

#### 3.1.2. ATP Dependent Microtubule Motor Activity

An ATP-dependent microtubule motor activity term (GO ID: HSA1990939) was enriched in vitrified human spermatozoa; the relevant gene heatmap shows that the expression level of the enriched genes consistently changed on the microtubule motor activity pathway (Figure 2B). The biological process in the GO database is shown in Figure 2C. The result shows the potential harm of mitochondrial dysfunction on cryopreserved groups. It should be noted that the vitrified group was enriched into this term, but not the conventional freezing group.

#### 3.1.3. Male Meiotic Nuclear Division (GO ID:0007140)

The Male Meiotic Nuclear Division was enriched in vitrified human spermatozoa and positively correlated (Figure 3A); the relevant gene heatmap shows that the expression level of the enriched genes consistently changed (Figure 3B). It should be noted that the vitrified group was enriched into this term, but not the conventional freezing group.

### 3.2. Ultrastructure Difference in Three Groups of Spermatozoa

**Figure 4:**
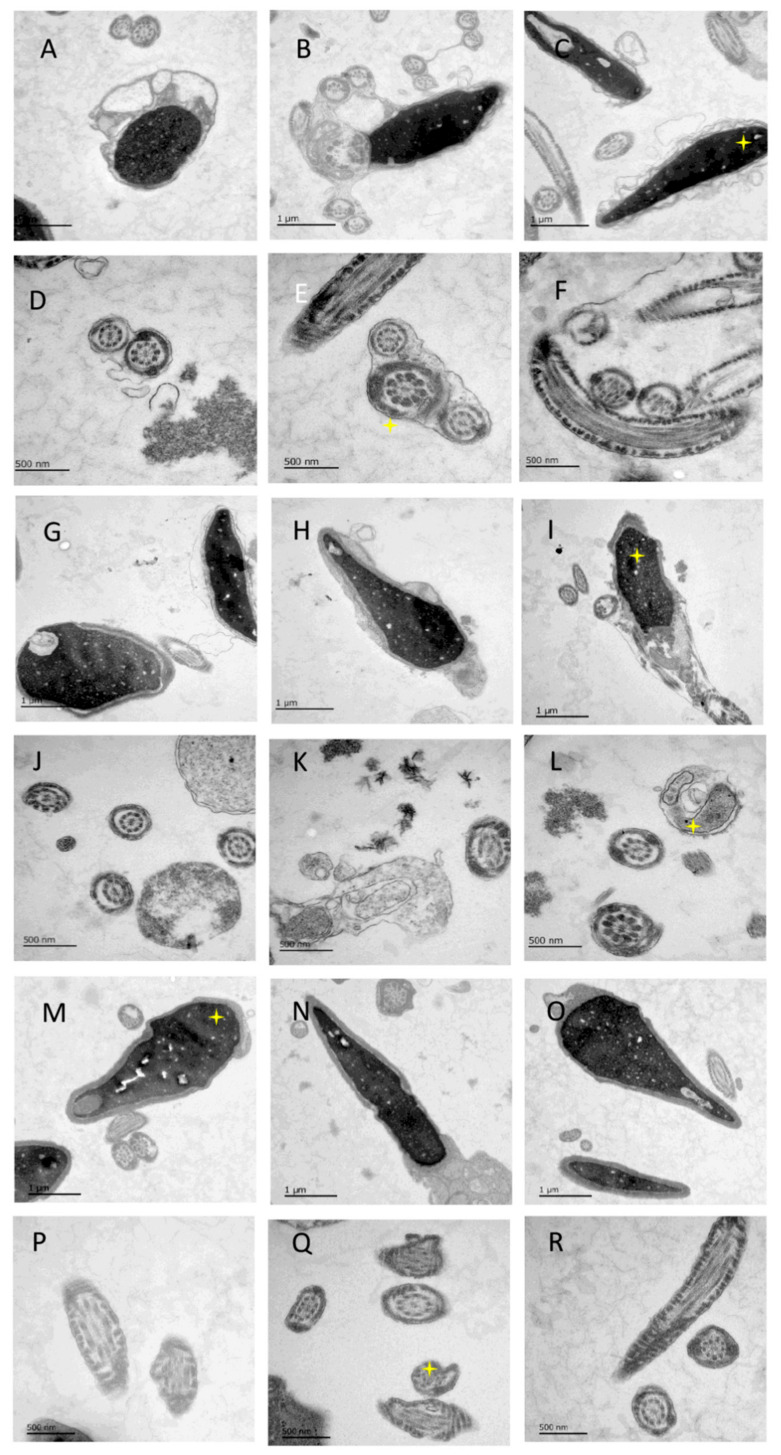
Ultrastructure of spermatozoa heads and mitochondria in fresh, slow-frozen, and vitrified samples. (**A**–**C**) Spermatozoa in fresh condition with clear edge of head; (**D**–**F**) normal mitochondria with dense structure in fresh spermatozoa group. (**G**–**I**) Photos of head in slow-frozen spermatozoa with swelling, disintegration, and disruption of plasma membrane of acrosome. (**J**–**L**) Photos of slow-frozen spermatozoa with loose mitochondrial structure, widening of mitochondrial cristae, and increased vacuole-like changes. (**M**–**O**) Photos of vitrified spermatozoa head with clear edge and complete head part. (**P**–**R**) Pictures of vitrified spermatozoa with dense mitochondrial structure, slightly lighter coloration, and widened mitochondrial cristae.

In Figure 4, the ultrastructure morphology shows spermatozoa in fresh conditions, they are with a clear edge of the head and normal mitochondria with a dense structure. A less empty vacuolated structure can be found in the fresh spermatozoa group (Figure 4A–F).

The heads of slow-freezing spermatozoa are with swelling, disintegration, and disruption of the plasma membrane of the acrosome. The mitochondrial structures are loose, and it is associated with the widening of mitochondrial cristae and increased vacuole-like changes.

Cryoprotectant-free vitrified spermatozoa heads have clear edges, complete structure, dense mitochondria, slightly lighter coloration, and widened mitochondrial cristae.

### 3.3. Alternative Splicing Event in Cryopreserved Spermatozoa

All detected AS events are shown in detail in the figshare database through a permanent DOI [43]. The files AS/SfvsFresh and AS/VivsFresh provide more informative results.

**Figure 5:**
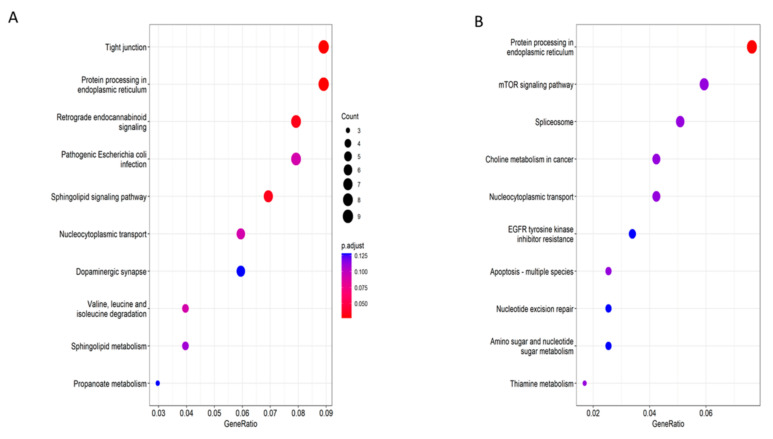
Visualization of KEGG pathway enrichment for alternative splicing events, especially exon skipping in two groups, ranked with inclusive difference value. (**A**) Enrichment results of exon skipping event with 10 significant terms (adjusted *p*-value < 0.05) in (**A**) cryoprotectant-free vitrified spermatozoa group and (**B**) conventionally frozen spermatozoa group.

As shown in Figure 5A, KEGG pathway enrichment revealed the frequent alternative splicing event. In the cryoprotectant-free vitrified spermatozoa group, there are exon skipping events in tight junction (hsa04530) and endoplasmic reticulum in protein processing (hsa04141), retrograde endocannabinoid signaling (hsa04723), and sphingolipid signaling pathway (hsa04071). As shown in Figure 5B, the conventionally frozen spermatozoa group also shows significance in protein processing in endoplasmic reticulum (hsa04141) and nucleocytoplasmic transport (hsa03013). The mTOR signaling pathway and spliceosome and nucleotide excision repair are also enriched terms.

#### Examples of A3SS, A5SS, MXE, SE and RI on Sequences

**Figure 6:**
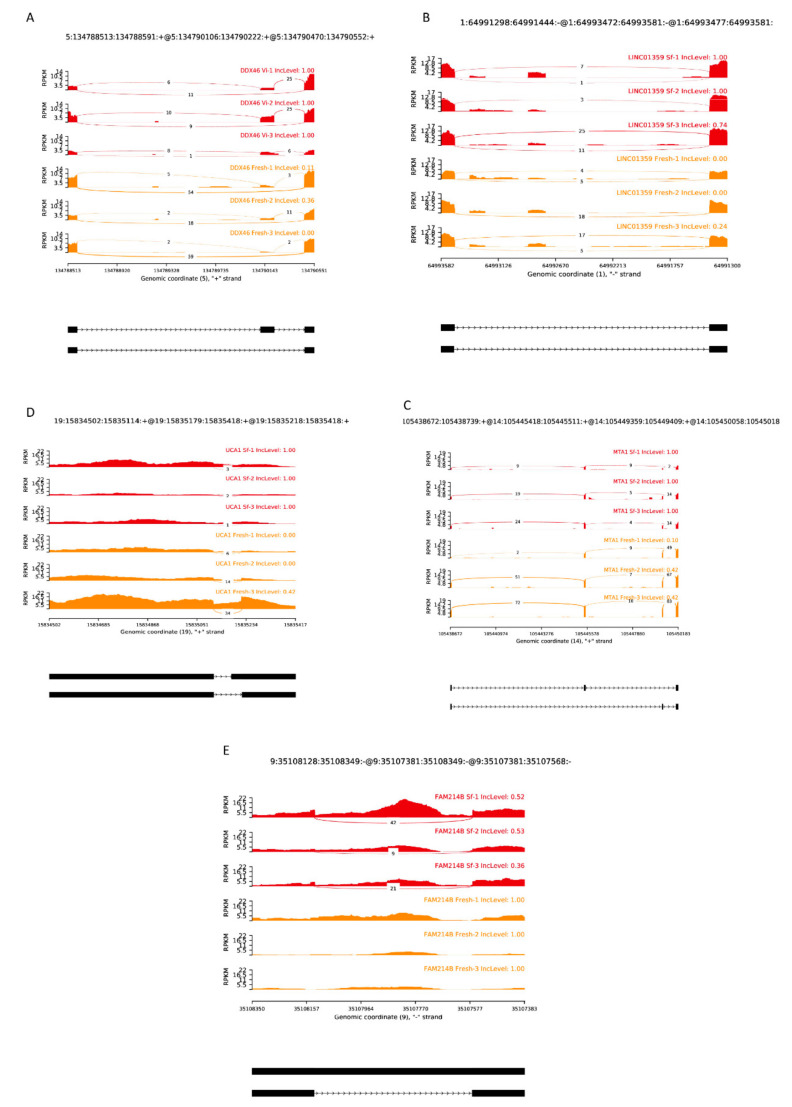
Examples of different types of alternative splicing events analyzed by rMATS. (**A**) Exon skipping diagram of *DDX46* in cryoprotectant-free vitrified spermatozoa group. (**B**) Alternative 5′ splice site graph of *LINC01359* in conventionally frozen spermatozoa group. (**C**) Mutually exclusive exon graph of *MTA1* in conventionally frozen spermatozoa group. (**D**) Alternative 3′ splice site graph of *UCA1* in conventionally frozen spermatozoa group. (**E**) Retained intron graph of *FAM214B* in conventionally frozen spermatozoa group.

The examples of different activity types in alternative splicing show how exons and introns have been spliced by these events (Figure 6). These functional genes are spliced with unknown and potentially harmful sites, which probably leads to wrong protein translation and disease. More examples can be found in figshare in file AS/SfvsFresh/ [43].

## 4. Discussion

### 4.1. GSEA Is a Advanced Method for the Spermatozoa Sequencing Datasets

It is believed that the GSEA method for analyzing sperm sequencing data has broad application value and should become the mainstream method; in fact, most analyses of gamete sequencing now focus on DEGs. Because gametes such as sperm are products of meiosis, they are fundamentally different from somatic cells. GSEA helps us to better understand the high-throughput data of gametes.

### 4.2. Mitochondrial tRNA Aminoacylation in Human Spermatozoa

Mitochondrial aminoacyl-tRNA synthetase proteins are a group of nuclear-encoding enzymes that ensure the correct translation of genetic information by regulating the binding of amino acids to their cognate tRNAs [44]. Mutations in mitochondrial aminoacyl-tRNA synthetase proteins frequently lead to clinically heterogeneous diseases, especially of the central nervous system [45]. There are tRNA aminoacylation-relevant genes correlated with cryoprotectant-free vitrified spermatozoa. For example, *NARS2* is the mitochondrial asparaginyl-tRNA synthetase relevant to human hearing loss and Leigh syndrome [46]. Alanyl-tRNA synthetase 2, with a mitochondrial (*AARS2*) mutation, has been reported in primary ovarian insufficiency, which can lead to abnormal aminoacylation of tRNA, affecting the process of mitochondrial translation and eventually leading to the occurrence of ovarian leukodystrophy syndrome [47]. Mutations in histidyl-tRNA synthetase 2, mitochondrial (*HARS2*), and leucyl-tRNA synthetase 2, mitochondrial (*LARS2*), are frequently associated with Perrault syndrome, which often leads to neuropathic hearing impairment and ovarian dysfunction in women [48,49]. However, there are no reports on mitochondrial tRNA aminoacylation affecting the male reproductive system.

### 4.3. ATP Dependent Microtubule Motor Activity

Kinesins are a superfamily that influence microtubule motility, and the core of their motor domain is a central beta-sheet and three alpha-helices on the left and right [50]. Kinesins convert their chemical energy into mechanical energy for movement along microtubules by binding to ATP [51]. Spermatozoa development requires the movement of microtubules to drive the assembly of the flagella on the spermatozoa head. For example, *KIF3A* and *KIF3B* may be involved in the formation of spermatozoa acrosomes, and silencing *KIF3B* can lead to defects in spermatozoa motility and morphology [52,53,54]. In turn, *KIF11* plays an important role in the travel of the spindle during spermatogonia meiosis [55]. Based on vitrification and enrichment analysis of fresh tissues, the kinesin family may indirectly affect spermatozoa motility in cryopreservation by affecting the motility of microtubules.

### 4.4. Male Meiotic Nuclear Division

In our results, the meiotic nuclear division term was active regarding RA*D51C* and so on; there was a similar trend with a possible late role in meiotic recombination in a mouse model [56].

### 4.5. Frequent Alternative Splicing Events, Especially Exon Skipping

Almost all human genes involve one or more alternative splicing events during transcription, including exon skipping, alternative 5′ splice sites, alternative 3′ splice sites, mutually exclusive exons, and retained introns [57]. The outcome of alternative splicing is influenced by cis-regulatory sequences, trans-acting factors including RNA-binding proteins (RBP) and splicing factors, and splice site strength [58]. The positive or negative regulation of the splicing process by the same RBP depends on the binding of the RBP to different regions of alternative splicing during transcription. Transcriptome analysis has shown that gene expression in testicular tissue significantly outnumbers that in other tissues, indicating that testis is a tissue with higher transcriptional complexity, including many genes involved in everything from mitosis of spermatogonia to meiosis of spermatozoa splicing transition [59]. Among them, *PTBP1* and *PTBP2* are differentially expressed in different stages of germ cell development. *PTBP1* is highly expressed in spermatogonia during mitosis, while *PTBP2* is significantly upregulated during spermatocyte meiosis. Abnormal changes in these two genes can lead to abnormal spermatogenesis [60,61]. For the first time, we have identified in human spermatozoa samples a splice variant of *DDX46*, a key gene in reproductive function regulation, which is generally thought to affect type I interferons and related pro-inflammatory cytokines by recruiting m6A “eraser” *ALKBH5* [62,63].

Our findings suggest that key fertility-related genes are significantly altered during cryopreservation, although it is unclear whether the alternative splicing behavior actually leads to differences in functional protein translation outcomes, including tight junction and endoplasmic reticulum. It was shown that inversin knockdown perturbed the tight Sertoli cell junction–barrier function in vitro and in vivo using corresponding physiological and integrity assays [64]. It is important to note that the endoplasmic reticulum plays an important role in spermatozoa maturation [65,66]. This requires more attention from cryobiologists.

### 4.6. Other Epigenetics Alternation in Human Gametes and Zygotes

The cryopreservation process has been known to cause epigenetic changes in spermatozoa, including DNA methylation, histone modification, and minor RNA downregulation [2,67]. Cryopreservation of spermatozoa, such as *Vasa* and *cxcr4b*, increases the degree of methylation [68]. For histone modification, spermatozoa histone H1–DNA binding and protein–DNA disulfide bonds were altered after cryopreservation [69]. The expression level of histone 4 was increased, suggesting that it may be associated with chromatin remodeling and compaction [70]. Micro-RNA affects mRNA translation, and *miR-22* and *miR-450b-5p* are significantly downregulated in spermatozoa after cryopreservation compared to fresh spermatozoa [10]. These epigenetic changes may be the main reason for the decline in spermatozoa motility and fertility affected by the cryo-thawing process.

### 4.7. Potential Modeling of Asthenozoospermia Using Vitrified Human Spermatozoa

The various splicing events and mitochondrial function changes found in this study can also be used as a model for asthenozoospermia [71]. Thus, sperm deficiency can be understood by studying changes in sperm cryopreservation. Meanwhile, the epigenetic differences found in the cryopreserved group in this study may also explain some of the pathophysiological processes of spermatozoa. For example, among the differences found in the cryopreserved group, mutations such as *DNAH1* and *DNAH17* lead to multiple abnormalities in flagella, which were found in both asthenozoospermic patients and the sequencing results of this study [72,73,74,75]. Second, the cryopreservation process often causes a certain degree of decreased motility and death of spermatozoa, which is a perfect model for studying asthenozoospermia because it provides a complete control and experimental group. It is possible that this also appears in sperm samples with asthenozoospermia [35]. In addition, our vitrification approach is safer without the addition of an osmotic protectant such as DMSO, whereas low concentrations of DMSO have been reported to induce dramatic changes in the epigenetic landscape of human gametes and embryos in vitro [16].

### 4.8. Limitation of the Study

Due to the small sample size, this study may still suffer from selection bias. Due to the specificity of the samples, only so many samples can be collected by existing means, thus all experimental methods were repeated three times. The problem of sample size has always been an objective problem in related research of human gametes. For example, some sperm proteomics studies can only detect single-digit samples [76,77], and sperm transcriptome analysis also has objective limitations.

## 5. Conclusions

Cryopreservation of spermatozoa from some patients can agitate epigenetic instability, including increased alternative splicing events and changes in crucial mitochondrial functional activities. For fertilization of oocytes, for such patients, it is recommended to use fresh spermatozoa whenever possible; cryopreservation of sperm is recommended to be used only in uncontested situations.

## Data Availability

The raw data of RNA-seq can be downloaded at “Sequence read archive” on National Center for Biotechnology Information. BioProject ID: PRJNA814701 (http://www.ncbi.nlm.nih.gov/bioproject/814701, accessed date: 6 April 2022). The metadata of GSEA and AS can be found in Figshare with permanent DOI:10.6084/m9.figshare.19576072.

## Supplementary Materials

The following supporting information can be downloaded at: https://www.mdpi.com/article/10.3390/cells11132110/s1, Figure S1. Schematic diagram of safety issues involved in human spermatozoa cryopreservation technology. Figure S2. Flowchart of study methodology. KEGG was used to enrich data twice, first with GSEA-KEGG, then with exon skipping-KEGG. These are different areas of study and add different elements to our knowledge. Figure S3. Gene Ontology enrichment plot (GO ID: HSA00970) for mitochondrial tRNA aminoacylation biosynthesis in cryoprotectant-free vitrified spermatozoa group
