# Supplementary material for: Epigenetic Alterations in Cryopreserved Human Spermatozoa: Suspected Potential Functional Defects
Source: Cells. 2022 Jul 4;11(13):2110. doi: 10.3390/cells11132110 (PMC9266127; doi:10.3390/cells11132110)
Supplement: Supplementary file 1 [file cells-11-02110-s001.zip › cells-1762587-supplementary.pdf]

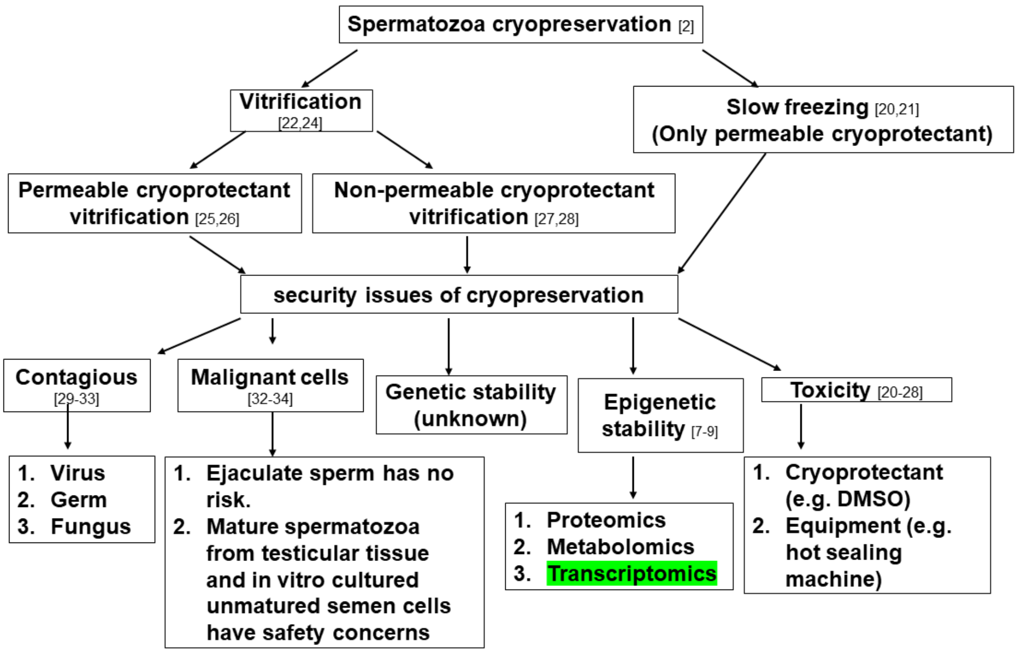

Figure S1. Schematic diagram of safety issues involved in human spermatozoa cryopreservation technology

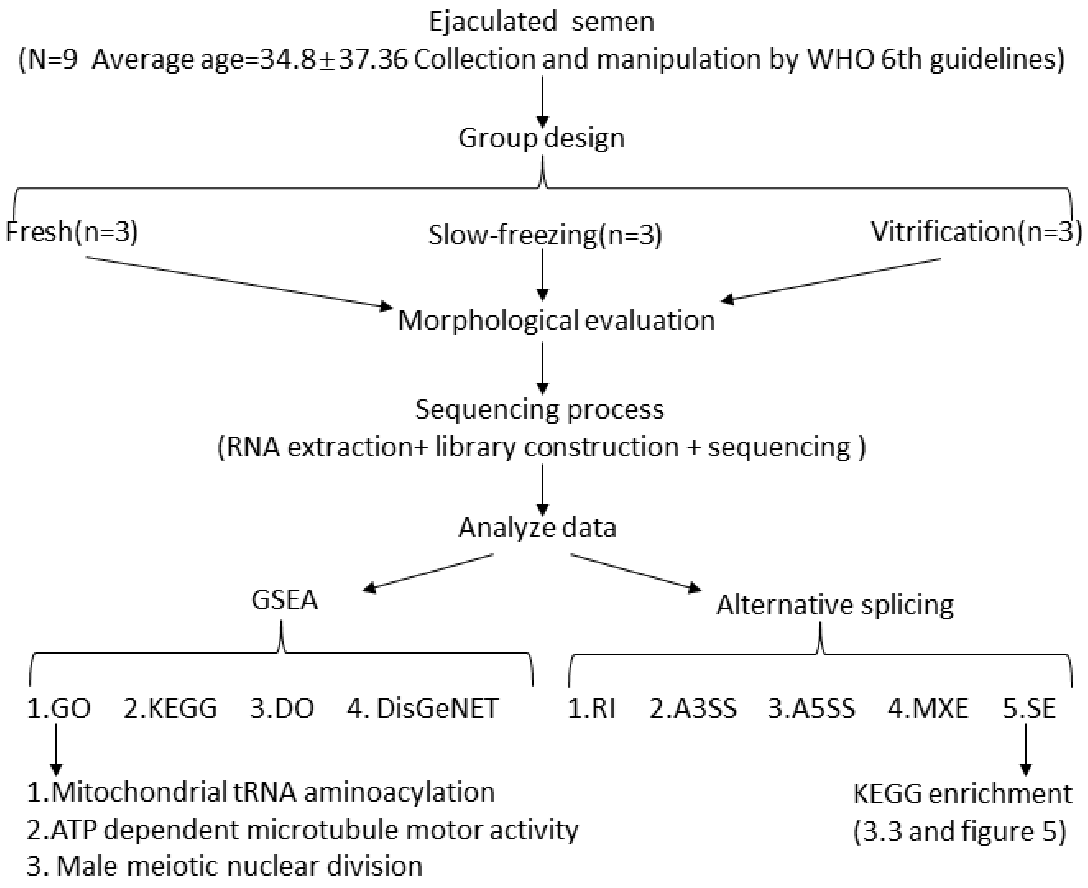

Figure S2. Flowchart of study methodology. KEGG was used to enrich data twice, first with GSEA-KEGG, then with exon skipping-KEGG. These are different areas of study and add different elements to our knowledge.

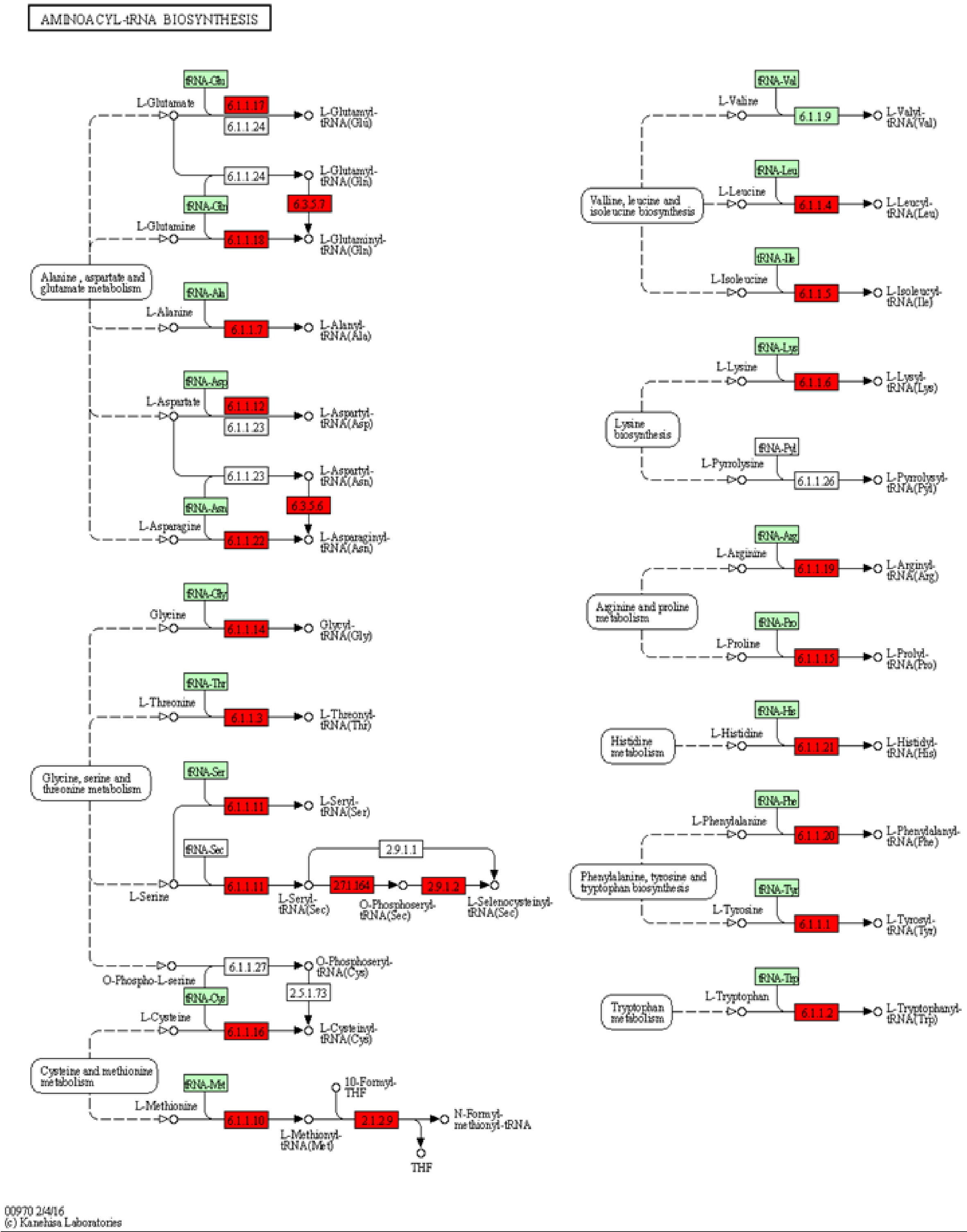

Figure S3. Gene Ontology enrichment plot (GO ID: HSA00970) for mitochondrial tRNA aminoacylation biosynthesis in cryoprotectant-free vitrified spermatozoa group
